# The Prognostic Role of Global Longitudinal Strain and NT-proBNP in Heart Failure Patients Receiving Cardiac Resynchronization Therapy

**DOI:** 10.3390/jpm14020188

**Published:** 2024-02-08

**Authors:** Nikolaos P. E. Kadoglou, Sjoerd Bouwmeester, Anouk G. W. de Lepper, Marloes C. de Kleijn, Ingeborg H. F. Herold, Arthur R. A. Bouwman, Ioannis Korakianitis, Tim Simmers, Franke A. L. E. Bracke, Patrick Houthuizen

**Affiliations:** 1 Medical School, University of Cyprus, 2029 Nicosia, Cyprus; 2 Department of Cardiology, Catharina Hospital Eindhoven, 5623 Eindhoven, The Netherlands

**Keywords:** cardiac resynchronization therapy—CRT, global longitudinal strain—GLS, natriuretic peptides, heart failure

## Abstract

Background: We aimed to evaluate whether baseline GLS (global longitudinal strain), NT-proBNP, and changes in these after cardiac resynchronization therapy (CRT) can predict long-term clinical outcomes and the echocardiographic-based response to CRT (defined by 15% relative reduction in left ventricular end-systolic volume). Methods: We enrolled 143 patients with stable ischemic heart failure (HF) undergoing CRT-D implantation. NT-proBNP and echocardiography were obtained before and 6 months after. The patients were followed up (median: 58 months) for HF-related deaths and/or HF hospitalizations (primary endpoint) or HF-related deaths (secondary endpoint). Results: A total of 84 patients achieved the primary and 53 the secondary endpoint, while 104 patients were considered CRT responders and 39 non-responders. At baseline, event-free patients had higher absolute GLS values (*p* < 0.001) and lower NT-proBNP serum levels (*p* < 0001) than those achieving the primary endpoint. A similar pattern was observed in favor of CRT responders vs. non-responders. On Cox regression analysis, baseline absolute GLS value (HR = 0.77; 95% CI, 0.51–1.91; *p* = 0.002) was beneficially associated with lower primary endpoint incidence, while baseline NT-proBNP levels (HR = 1.55; 95% CI, 1.43–2.01; *p* = 0.002) and diabetes presence (HR = 1.27; 95% CI, 1.12–1.98; *p* = 0.003) were related to higher primary endpoint incidence. Conclusions: In HF patients undergoing CRT-D, baseline GLS and NT-proBNP concentrations may serve as prognostic factors, while they may predict the echocardiographic-based response to CRT.

## 1. Introduction

Patients with symptomatic (NYHA II–IV) heart failure (HF), reduced left ventricular ejection fraction (LVEF), and wide QRS complex are good candidates for cardiac resynchronization therapy (CRT), since it is associated with significant improvements in symptoms and morbidity and mortality (level of evidence IA or IIaB) [1,2]. Unfortunately, a significant proportion of CRT recipients (~30%) do not experience the expected clinical benefits, despite the fulfillment of classical selection criteria [3]. That proportion is almost similar between registries, despite the application of several approaches. The characteristics of those patients, so-called non-responders, have been put under investigation in order to maximize the efficacy of this costly therapy. The site of LV electrode implantation and the post-intervention management may determine the response to CRT in a predictable way, but the most challenging parameter remains patient selection [4]. Although the response to CRT is clinically judged, which requires a long-term clinical follow-up, there is a growing body of evidence investigating the echocardiographic-based response, a surrogate marker of clinical response [5]. Over and above other indices, a significant reduction (by ≥15%) in LV end-systolic volume (LVESV) has been proposed as an echocardiographic-derived index of effective response to CRT-D implantation [6]. Patients achieving the aforementioned ESV decline are considered “CRT responders”. That change requires 6 months to become recognizable, and most but not all previous studies have shown a significant association with clinical outcomes in CRT-D receivers, supporting its application as a surrogate prognostic factor [7,8]. This is an attempt to shorten the follow-up time and facilitate the early recognition of HF patients who will not derive advantage from CRT (non-responders) who should search for optimum medical therapy.

Regarding this gap, several investigators have proposed supplementary echocardiographic indices to predict clinical response to CRT [9]. Global longitudinal strain (GLS), an index quantifying active global myocardial deformation, has commonly been reported as a valid predictor of CRT response [10]. In particular, a high absolute baseline GLS and the amount of its change post-intervention have been related to favorable clinical outcomes of CRT in HF patients. Most, but not all, studies have supported the prognostic power of GLS in CRT recipients [11]. However, there is still insufficient evidence to support its use as a single prognostic factor.

Biomarkers have also been proposed as predictors of clinical outcomes in patients with HF [12]. A growing body of evidence supports its usage for tailoring CRT implantation and candidate selection [13]. Among those biomarkers, N-terminal prohormone of B-type natriuretic peptide (NT-proBNP) has emerged as the most promising prognostic factors, but the available data about its application in CRT guidance are limited and controversial [14,15].

The aim of the present study was to investigate whether the baseline values of GLS and NT-proBNP and/or changes in these after implantation in patients with HF fulfilling the criteria for CRT can predict: (1) long-term morbidity and mortality and (2) response to CRT-D based on echocardiographic criteria. For comparison reasons, we examined our cohort using either clinical criteria (event-free patients versus patients with at least one event (hospitalization and/or death)) or echocardiographic criteria (CRT responders versus CRT non-responders).

## 2. Materials and Methods

### 2.1. Study Design

This was a prospective, observational study conducted in two hospitals from 2016 to 2019. We initially recruited 147 patients with ischemic HF (NYHA class II/III, ejection fraction ≤ 35%) referred for CRT-D according to the international criteria and despite the already optimum HF therapy [16]. The ischemic etiology was defined by the history of myocardial infarction and/or coronary revascularization procedure (percutaneous coronary angioplasty, coronary artery bypass grafting). At baseline and before CRT-D implantation, all patients underwent initial evaluation, including medical history, NYHA class assessment, blood pressure, blood sampling for NT-proBNP assay and echocardiographic examination. That evaluation was repeated 6 months after implantation by the same cardiologists. After the second assessment, we continued follow-up of all alive participants by retrieving records from the national health-care operation systems of our countries or hospital records, or when they were not available, by telephone interview of patients or their siblings. In cases of patient death, we searched for medical records to clarify the cause of death and endpoint achievement.

Based on echocardiographic criteria, patients presenting ≥ 15% reduction in their LVESV from baseline to 6 months were considered “CRT responders” and the rest “CRT non-responders”. During the whole study period, we evaluated the achievement of the primary endpoints of HF-related deaths and/or hospitalizations and secondary endpoint of HF-related deaths. We searched for other endpoints, like all-cause mortality, myocardial infarction, or cardiovascular morbidity, but we realized that the low number of recorded events prevented us from any further analysis or were overlapped by other events. Among all potential prognostic factors, we evaluated baseline GLS and NT-proBNP and changes in these after implantation in patients with HF undergoing CRT-D.

Informed consent was obtained from all subjects involved in the study.

### 2.2. Echocardiography

Using a standard imaging system (iE33 ultrasound scanner equipped with a S5-1 transducer (Philips Healthcare, Andover, MA, USA)), two independent operators performed the echocardiographic studies at baseline and 6 months after implantation. The offline imaging analysis was performed on a single PC workstation by two cardiologists blinded to patients’ data. Standard echocardiographic measurements included LVEF, LV end-diastolic volume (LVEDV) and LVESV.

Speckle tracking echocardiography has been developed for the assessment of myocardial deformation. The motion of speckles in a certain region of interest is traced frame by frame throughout the cardiac cycle. In a normal individual, the strain curves reach its peak strain at peak systole in a coordinated manner. GLS has been recommended as an index of speckle tracking echocardiography with high accuracy for the quantification of LV systolic dysfunction [17]. We acquired all images at the maximum possible frame rates using the same vendor for image analysis. GLS was derived from speckle tracking and analyzed by post-processing of all 3 apical images of the LV (4-chamber, 2-chamber, 3-chamber). In all the three apical images, the echocardiography software divided each wall into 3 segments in which peak systolic longitudinal strain values were calculated. Then, the mean peak longitudinal strain of each imaging plane and the mean of the peak global longitudinal strain values from all segments were obtained. A GLS value < −18% usually falls within the normal range, while an increasing GLS from >−16% parallels the degree of LV systolic dysfunction.

### 2.3. Statistical Analysis

Data are expressed as means ± SD. Normality of distribution was assessed by Kolmogorov–Smirnov test. Based on primary endpoints, we divided patients into event-free and those experiencing an event. Based on echocardiographic criterion of CRT response, we compared CRT responders with non-responders. Paired-sample t-tests and Student’s t-tests were used for comparison of continuous parameters within and between groups, respectively. Differences between groups were assessed using the Mann–Whitney U test for variables with non-normal distribution and the χ^2^ test for categorical variables as appropriate. Paired sample t-tests were used to compare changes in echocardiographic parameters from baseline to follow-up. Cox regression analysis was used for prediction of the primary and secondary clinical endpoints. Hazard ratios (HRs) and 95% confidence intervals (CIs) were calculated for each factor via Cox proportional hazard analysis. All baseline variables with *p* < 0.05 on univariate analyses were integrated into the Cox multivariate model to determine the independent predictors of primary and secondary endpoints. Logistic regression analysis was used to assess the association of baseline variables with echocardiographic-based CRT response at 6 months after implantation. Survival analysis using the Kaplan–Meier method with log-rank test was used to analyze the cumulative survival starting from baseline. Receiver-operating characteristic (ROC) curves were used to assess the diagnostic accuracy for discriminating the achievement of endpoints or not. Area under the curve (AUC), sensitivity, and specificity were calculated. Two-tailed *p* values < 0.05 were defined as statistically significant. We used the statistical software SPSS-25.0 (SPSS Inc, Chicago, IL, USA) for analyses.

## 3. Results

### 3.1. Groups at Baseline

Overall, patients initially entered analysis. We further excluded two patients, who were lost during follow-up for personal reasons and another two patients, who died just after CRT-D implantation and before discharge due to infection and acute heart failure, respectively.

We obtained full data from 143 patients. During a median follow-up of 58 months, 84 patients achieved the primary endpoint (death and/or hospitalization due to HF). The group achieving the primary endpoint was a substantial expansion of that of the secondary one. Notably, almost all patients before HF-related death had at least one hospitalization for HF decompensation. In the whole cohort at baseline, 56 patients died from any cause. Among them, 53 deaths were attributed to HF (secondary endpoint), while 3 patients died due to other reasons: myocardial infarction, newly diagnosed lung cancer and car accident. Hence, the all-cause mortality was predominantly driven by HF-related mortality.

### 3.2. Prognostic Value of GLS and NT-proBNP at Baseline

**Figure 1:**
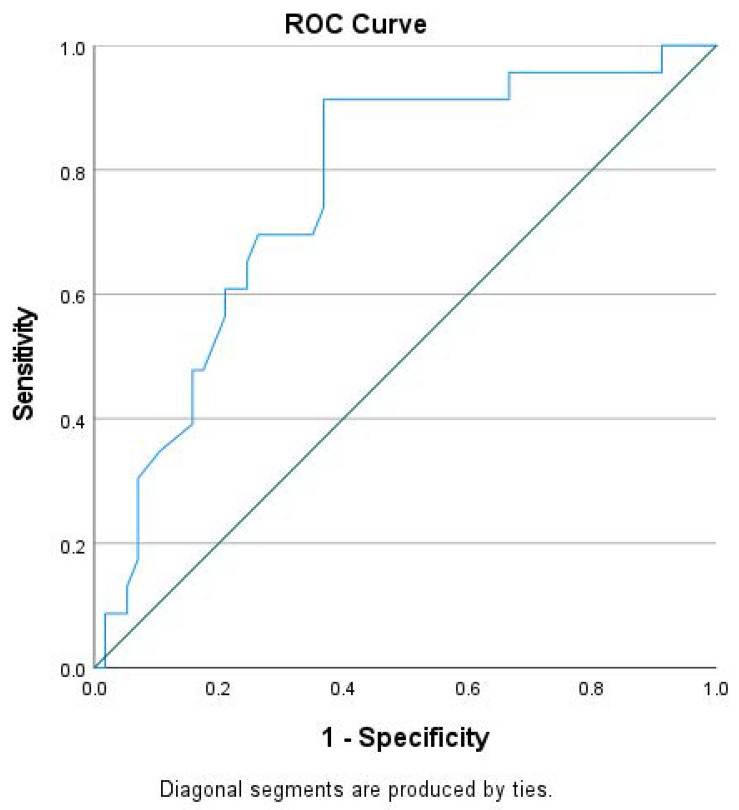
Receiver-operating characteristic (ROC) curve of global longitudinal strain (GLS) for the prediction of the primary endpoint (HF-related deaths and/or hospitalizations).

Based on clinical endpoints, we assessed the prognostic value of GLS and NT-proBNP. Considering that hospitalization for HF preceded almost all HF-related deaths, we paid attention to the group achieving the primary endpoint. That group showed higher NT-proBNP and lower absolute GLS values at baseline compared to the event-free group (Table 1). Notably, those groups did not differ in other significant parameters, like most demographic characteristics, QRS duration, pharmaceutical therapy etc. On Cox regression analysis, lower GLS absolute value, higher NT-proBNP and the presence of diabetes mellitus at baseline were associated with higher incidence of death and/or hospitalization due to HF on multivariate analysis (Table 2). In our cohort, using the ROC analysis, we found that GLS had an area under the curve of 0.75, which gave a cutoff value of GLS > −7.92% to predict the occurrence of primary endpoint with very modest sensitivity (69.0%) and specificity (70.4%) (Figure 1).

With a smaller group achieving it, baseline NT-proBNP, along with atrial fibrillation and kidney dysfunction, emerged as independent predictors of the secondary endpoint (Table 3).

### 3.3. Prediction of CRT Response by GLS and NT-proBNP

**Figure 2:**
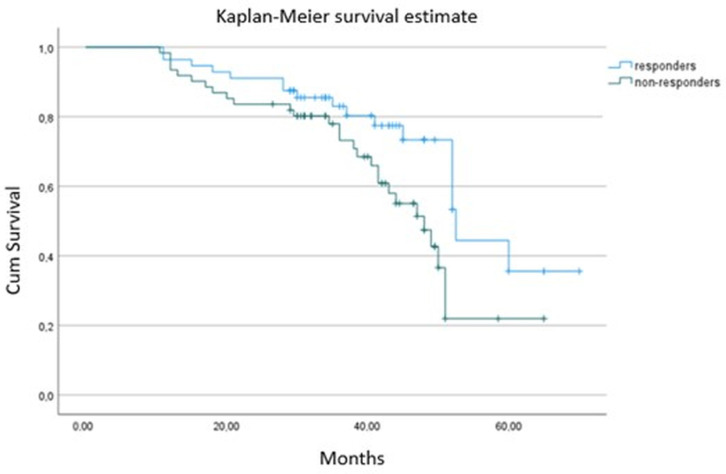
Kaplan–Meier survival curve between responders and non-responders from 6 months to the end of follow-up.

As expected, CRT responders had better survival and experienced fewer hospitalizations (*p* < 0.001) compared to non-responders (Table 4 and Figure 2). At baseline, CRT responders showed higher absolute GLS values (*p* < 0.001), larger left atrium volumes (*p* = 0.029) and lower NT-proBNP serum levels (*p* < 0001) than non-responders. There was a marginal, non-significant difference between groups in QRS duration (*p* = 0.061), with responders having a non-significantly broader QRS complex. There were no significant differences in the rest of the clinical, pharmaceutical or echocardiographic parameters (*p* > 0.05) (Table 4). Although CRT responders had higher absolute baseline GLS values and lower NT-proBNP levels, we failed to find any independent association of either absolute GLS (OR = 1.11; 95% CI, 1.01–1.32; *p* = 0.660) or NT-proBNP (OR = 1.35; 95% CI, 1.19–1.75; *p* = 0.088,) at baseline with CRT response at 6 months on logistic regression analysis.

### 3.4. Changes in GLS and NT-proBNP with CRT and Their Prognostic Value

Regarding the prognostic value of the CRT-induced changes in GLS and NT-proBNP in the 6-month post-intervention period, the event-free group had a larger increase in absolute GLS (ΔGLS: 4.1 ± 0.8% vs. 0.55 ± 0.3%, *p* < 0.001) and remarkable decrease in NT-proBNP levels (ΔNT-proBNP: −588 ± 281 pg/mL vs. −101 ± 78 pg/mL, *p* = 0.003) than those who had at least one hospitalization.

**Figure 3:**
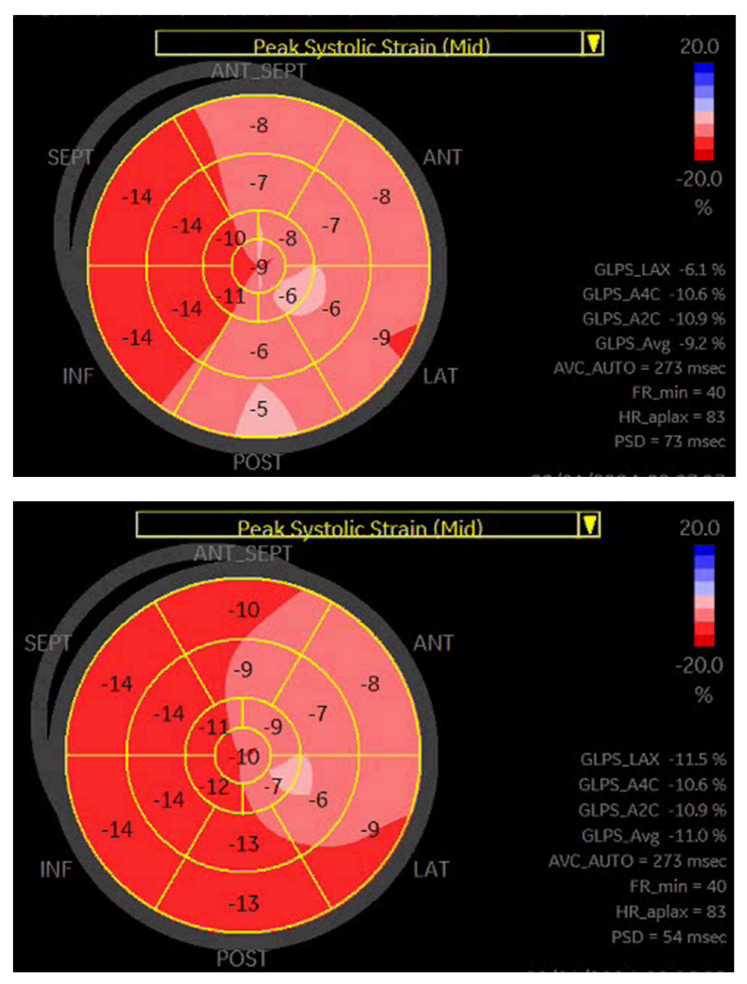
A representative example of GLS improvement in a patient after CRT-D implantation.

Based on a previous publication [18], we performed a subgroup analysis looking at patients with >25% reduction in NT-proBNP concentration and concomitant > 30% improvement in GLS. All patients fulfilling those criteria belonged to the responder group (*n* = 45). Notably, compared to the remaining responders (*n* = 59), the mortality rate was comparable; however, fewer patients achieved the primary endpoint during follow-up (7 versus 24, *p* < 0.001). Figure 3 presents an example of GLS improvement in a CRT responder.

## 4. Discussion

In the present prospective, observational study, the low baseline pre-interventional values of both absolute GLS and NT-proBNP independently predicted the primary clinical endpoint, while low circulating NT-proBNP levels independently predicted the secondary clinical endpoint in the long-term follow-up (almost 6 years). On the other hand, the higher absolute GLS and lower NT-proBNP levels at baseline failed to predict the echocardiographic-based CRT response. Within 6 months of CRT-D implantation, both event-free patients and CRT-D responders had significantly ameliorated GLS and NT-proBNP levels, but that effect did not determine prognosis.

The identification of patients who may not benefit from CRT implantation (non-responders) using feasible and easily assessed parameters is of crucial importance. Several factors have been recommended, but no single factor has been specifically powered to identify potential non-responders and guide the exclusion of them prior to CRT. Shorter QRS duration (<130 ms), an ischemic origin of HF, male gender and non-LBBB pattern have been proposed as negative predictors of CRT clinical response [19]. Considering that LV dyssynchrony is the target of CRT, several conventional echocardiographic indices with potential prognostic value have been proposed, but the long-term results are very modest and not yet clinically applicable [20]. The assessment of LV deformation using GLS seems to be a good tool to adequately predict and monitor the response to CRT device implantation [21]. The echocardiographic response to CRT has been associated with clinical improvement and reduced morbidity and mortality [22]. Most, but not all, researchers have demonstrated a strong association of a significant decrease in ESV after CRT implantation with beneficial outcomes in the long term, proposing it as a surrogate marker of clinical response to CRT. An advantage of using surrogate markers is an early and highly accurate estimation of a therapy’s efficacy before the occurrence of any cardiovascular event. From the methodological point of view, this study evaluated the relationship of baseline GLS and NT-proBNP with CRT response using both criteria: clinical outcomes during follow-up and the echocardiographic-based index. Our findings are consistent with previous studies reporting higher absolute value of GLS in responders than non-responders defined by both criteria [23]. A recent meta-analysis using either clinical or echocardiographic characteristics of CRT response confirmed better resting GLS values in CRT responders than non-responders (Bazoukis et al. 2020) [8]. That meta-analysis used a mixed definition of CRT response, which may be confounding. Presumably, a higher absolute GLS at baseline expresses less burden of non-viable, scarred myocardium, with potentially higher response rate. Therefore, the elevated absolute GLS at baseline predisposes more likely to echocardiographic-based CRT response. However, neither specific cutoff values of GLS have been provided nor an independent relationship between GLS and beneficial changes in ESV after CRT have been consistently reported. Importantly, in our study, we failed to demonstrate an independent relationship of those parameters with echocardiographic-based response, raising questions about their validity.

The conventional echocardiographic-defined CRT response consists of a surrogate marker, but it may not entirely reflect the clinical course of CRT receivers. From the clinical perspective, the prognostic validation of markers such as GLS or others should take place in relation to clinical outcomes. Surprisingly a small number of studies, all from the same research center, have confirmed a direct relationship of baseline GLS with clinical outcomes [6,24,25]. In our study, the event-free patients had higher absolute GLS levels at baseline, implicating an indirect relationship between variables. Most importantly, we demonstrated a direct independent relationship of GLS with the combined clinical endpoint (death and HF hospitalization) on Cox regression analysis. Our study supports the application of GLS for the detection of patients who may get benefit from CRT device implantation affecting the decision-making process. The clinical relevance should be further investigated, because in multivariate models, the characteristics of studied cohorts, the homogeneity, and the power of the study may determine the independent relationship of any variable within groups. On the other hand, the relationship of GLS with clinical endpoints in HF patients not requiring CRT has long been demonstrated [26]. Despite accumulated data, there is not yet a widely accepted cutoff value for either resting GLS or GLS change to predict clinical response. Based on ROC analysis, we selected a cutoff value of absolute GLS > −7.92% for the prediction of the combined endpoint in CRT receivers, but with very modest sensitivity and specificity. A single previous study has also proposed an almost equivalent discriminatory value of GLS for clinical outcomes, but it has not been validated yet [27].

Despite the remarkable difference in GLS between CRT responders and non-responders, we failed to find an independent association of baseline GLS with a change in ESV values at 6 months. Contrary to a previous study, we did not confirm the prognostic value of echocardiographic-based CRT response and hence its relationship with GLS [10]. Perhaps GLS may not be able to serve as a predictor of another echocardiographic index (ESV change) in the selected timeframe of 6 months or in our cohort with specific characteristics. This raises questions about the clinical applicability of echocardiographic-based CRT response. In parallel, we demonstrated that CRT responders showed improved GLS to a larger extent than non-responders, which agrees with previous meta-analyses. This is an expected result, but the percentage of GLS change that could predict clinical improvement and survival has not yet been determined. Therefore, more studies are required to investigate the clinical impact of GLS in decision-making in patients undergoing CRT.

To our knowledge, this is the second study demonstrating the inverse relationship of NT-proBNP at baseline with favorable clinical outcomes in the long term and CRT response. The CARE-HF trial, with extensions of follow-up, was the first and largest trial supporting the prognostic value of NT-proBNP in CRT receivers [28,29]. A growing body of evidence implicates the prognostic power of NT-proBNP, which may significantly influence future therapeutic decisions in HF [30]. Therefore, NT-proBNP may become a significant biomarker predicting the efficacy of CRT and identifying candidates, who will gain benefits from this interventional therapy [31]. Despite those promising results, there are some important limitations for widespread application of biomarkers [12]. First of all, this is a multifaceted biomarker, which may be affected by concomitant pharmaceutical therapy and comorbidities attenuating its prognostic value. Second, there is not yet a cutoff value to predict event-free clinical course or CRT response. Inversely, CRT favorably reduced NT-proBNP circulating levels in responders; however, changes in it alone did not have any prognostic value for CRT receivers. Nevertheless, NT-proBNP remains a cheap, well-established, easily measured biomarker with potential application for the detection of appropriate CRT candidates.

In agreement with previous reports, our CRT responders showed higher improvement in GLS compared to non-responders. Notably, patients with >30% increase in GLS concomitant with >25% reduction in NT-proBNP were all assigned to the responder group. Those patients had the best clinical course compared not only to non-responders but also to responders with less improvement in both GLS and NT-proBNP. Perhaps a combined approach of echocardiographic assessment of deformation and natriuretic peptides may further enhance the early discrimination of super-responders. Our study was not powered to quantify the clinical impact of such a combination.

Among the most important limitations of the present study were its relatively small sample and the short-term (6 months) monitoring of echocardiographic performance and NT-proBNP levels. We could not control parameters for the whole follow-up period, focusing solely on clinical endpoints. On the other hand, we recruited a more homogeneous cohort by involving patients with ischemic HF receiving only CRT-D. The threshold defining response to CRT is a matter of debate. It is predominantly based on echocardiography rather than clinical assessment and its calculation is vendor-dependent, so it may vary across different vendor platforms. Finally, no patient was on sacubitril–valsartan or SGLT2i, since the study was completed by the end of 2021, when the new guidelines were announced by the European Society of Cardiology.

In conclusion, lower absolute values of GLS and higher NT-proBNP levels predicted the primary endpoint of death and HF hospitalization in HF patients undergoing CRT-D and the echocardiographic-assessed non-response. Moreover, low baseline NT-proBNP was independently associated with survival in the long term. Finally, a significant amelioration of concomitant GLS and NT-proBNP after CRT-D implantation did not add prognostic value in our cohort.

## Figures and Tables

**Table 1:** Baseline characteristics of event-free patients compared to those achieving the primary endpoint (HF-related deaths and/or hospitalizations).

	No Events (*n* = 58)	Primary Endpoint (*n* = 84)	*p*
Age, years	71 ± 10	73 ± 9	0.578
Males, *n*	43 (73%)	60 (71%)	0.789
LVEF (%)	29 ± 9	26 ± 10	0.777
LVESV (mL)	142 ± 61	156 ± 54	0.403
LAVI (mL/m^2^)	34 ± 8	38 ± 8	0.122
GLS (%)	−8.8 ± 2	−6.1 ± 2.2	<0.001
QRS duration (ms)	157 ± 25	155 ± 21	0.061
NYHA II	18 (31%)	21 (25%)	0.411
NYHA III	40 (69%)	63 (75%)	0.173
Diabetes, *n*	12 (20.7%)	23 (27.4%)	0.025
Atrial fibrillation	9 (15.5%)	19 (22.6%)	0.101
CKD (stage IV–V), *n*	2 (3.4%)	4 (4.8%)	0.535
NT-proBNP (pg/mL)	1449 ± 288	2210 ± 420	<0.001
Diuretics, *n*	56	82	0.923
ACEIs/ARBs, *n*	50	64	0.875
MRA, *n*	44	62	0.813
β-blockers, *n*	53	78	0.819

ACEIs, angiotensin-converting enzyme inhibitors; ARBs, angiotensin receptor blockers; CKD, chronic kidney disease; GLS, global longitudinal strain; LAVI, left atrial volume index; LVEF, left-ventricular ejection fraction; LVESV, left-ventricular end-systolic volume; MRA, mineralocorticoid receptor antagonist; *n*, number.

**Table 2:** Uni- and multivariate Cox proportional hazard models of HF-related deaths and/or hospitalizations (primary endpoint).

Variable	Univariate		Multivariate	
	HR (95% CI)	*p*	HR (95% CI)	*p*
Age (years)	1.04 (1–1.08)	0.891		
Male gender	0.93 (0.77–1.13)	0.770		
NYHA	1.04 (0.95–1.18)	0.441		
QRS (ms)	1.15 (1.01–1.29)	0.126		
Diabetes mellitus	1.52 (1.28–2.05)	<0.001	1.27 (1.12–1.98)	0.003
Chronic kidney disease (eGFR < 45 mL/min/1.73 m^2^)	1.88 (1.39–2.74)	0.009	1.29 (1.10–2.12)	0.068
CRT response	1.30 (1.03–1.85)	0.033	1.12 (0.98–1.43)	0.394
GLS (absolute value %)	0.48 (0.32–2.1)	<0.001	0.77 (0.51–1.91)	0.002
NT-proBNP	1.78 (1.59–245)	<0.001	1.55 (1.43–2.01)	0.002

**Table 3:** Uni- and multi-variate Cox proportional hazard models of the secondary endpoint (HF-related deaths).

Variable	Univariate		Multivariate	
	HR (95% CI)	*p*	HR (95% CI)	*p*
Age (years)	1.01 (0.99–1.03)	0.932		
Male gender	1.05 (0.91–1.19)	0.690		
NYHA	1.11 (1.01–1.25)	0.702		
QRS (ms)	1.15 (1.01–1.29)	0.126		
Diabetes mellitus	1.28 (1.10–1.88)	0.008	1.27 (1.12–1.98)	0.087
Atrial fibrillation	1.89 (1.51–2.67)	<0.001	1.66 (1.31–2.22)	<0.001
Chronic kidney disease (eGFR < 45 mL/min/1.73 m^2^)	1.66 (1.33–2.55)	0.006	1.41 (1.17–1.98)	<0.001
CRT response	1.91 (1.23–3.05)	0.012	1.22 (1.15–2.43)	0.132
GLS (absolute value %)	0.67 (0.56–1.05)	0.015	0.96 (0.81–1.11)	0.091
NT-proBNP	1.64 (1.23–2.22)	<0.001	1.23 (1.01–1.69)	<0.001

**Table 4:** Characteristics of CRT responders and non-responders at baseline.

	CRT Responders (*n* = 104)	CRT Non-Responders (*n* = 39)	*p*
Age, years	70 ± 11	74 ± 7	0.423
Males, *n*	74 (71.2%)	29 (74.4%)	0.831
LVEF (%)	28 ± 6	26 ± 8	0.891
LVESV (mL)	148 ± 55	153 ± 52	0.790
LAVI (mL/m^2^)	37 ± 7	33 ± 5	0.029
GLS (%)	−8.2 ± 2.4	−6.2 ± 1.8	<0.001
QRS duration (ms)	167 ± 29	151 ± 22	0.061
NYHA II	31 (30%)	15 (38.5%)	0.309
NYHA III	73 (70.2%)	24 (61.5%)	0.298
Diabetes, *n*	25 (24%)	10 (25.6%)	0.925
Atrial fibrillation	21 (20.2%)	7 (17.9%)	0.881
CKD (stage IV–V), *n*	4 (7.7%)	2 (5.1%)	0.123
NT-proBNP (pg/mL)	1589 ± 232	1998 ± 308	<0.001
Diuretics, *n*	101	37	0.955
ACEIs/ARBs, *n*	88	32	0.906
MRA, *n*	74	32	0.881
β-blockers, *n*	95	36	0.984
Primary endpoint, *n*	52 (50%)	32 (82%)	<0.001
Secondary endpoint, *n*	35 (33.6%)	18 (46.2%)	0.007

ACEIs, angiotensin-converting enzyme inhibitors; ARBs, angiotensin receptor blockers; CKD, chronic kidney disease; GLS, global longitudinal strain; LAVI, left atrial volume index; LVEF, left-ventricular ejection fraction; LVESV, left-ventricular end-systolic volume; MRA, mineralocorticoid receptor antagonists *n*, number.

## Data Availability

Data are contained within the article.
